# Navigating the Road Ahead: A Scoping Review of Care Partner Involvement in Driving Decisions for People with Dementia

**DOI:** 10.1093/geroni/igaf122.2013

**Published:** 2025-12-31

**Authors:** K J Hansmann, Mark D’Alesio, Anthony Mcdonald, Beth Fields

**Affiliations:** University of Wisconsin–Madison, Madison, Wisconsin, United States; University of Pittsburgh Medical Center, Pittsburgh, Pennsylvania, United States; University of Wisconsin–Madison, Madison, Wisconsin, United States; University of Wisconsin–Madison, Madison, Wisconsin, United States

## Abstract

The prevalence of dementia is steadily increasing among older adults, raising concerns about safe driving as cognitive and functional decline impact the ability to drive safely. People with dementia often rely on care partners (e.g., family, friends) for support with daily activities and health care management due to the progressive nature of the disease. However, their role in driving-related decisions and transitions remains less understood. This scoping review aims to identify and characterize how care partners are involved in research on driving for people with dementia. We included articles if they (a) were full-text articles describing qualitative, quantitative, or mixed methods research; (b) investigated driving for people with dementia; and (c) directly involved care partners in the study. Multiple reviewers completed abstract and full-text screening, and completed a qualitative descriptive analysis to summarize themes drawn from the included articles. We identified 15 research articles that included care partners in studies investigating people with dementia’s driving. Of these, 12 specifically focused on driving cessation. Our analysis revealed three key themes describing care partners’ involvement: (1) sharing their emotional experiences related to driving restriction and cessation, (2) serving as decision-makers about driving restriction and cessation, and (3) providing insights into the driving behaviors of people with dementia. Care partner’s inclusion in driving research and interventions facilitates addressing safety concerns. In addition, care partner involvement in the research process can support the development of innovative approaches that extend beyond driving cessation to support broader community mobility needs for people with dementia.

